# Spontaneous Gastric Perforation in a Child and Endoscopic Balloon Dilation for Postoperative Gastric Stenosis

**DOI:** 10.1097/PG9.0000000000000199

**Published:** 2022-04-08

**Authors:** Bethany S. Cunningham, Chandran P. Alexander

**Affiliations:** From the Department of Pediatrics, Division of Pediatric Gastroenterology and Nutrition, Penn State Milton S Hershey Children’s Hospital, Hershey, PA.

In this issue of *JPGN Reports*, Peña-Vélez et al^1^ describe a young girl presenting with a spontaneous gastric perforation and who subsequently underwent >60% partial gastrectomy, intensive care, and nutritional rehabilitation. She was diagnosed to have polyarteritis nodosa (PAN) with left gastric artery thrombosis treated with immunosuppression and anticoagulation. At follow–up, a few months later, she had symptoms of gastric outlet obstruction (GOO) resulting in significant weight loss. Upper gastrointestinal contrast series and endoscopy confirmed severe gastric outlet stenosis. Three sequential graded through-the-scope (TTS) balloon dilations provided relief and helped this seriously ill child avoid another surgery.^1^

While both wire-guided fluoroscopic and TTS balloon dilations in patients with esophageal strictures and GOO from differing etiologies have been well documented for the past three decades,^2^ this report highlights its successful application in a postoperative gastric stricture, which is not often seen in children. No specific guidelines for TTS balloon dilations for GOO in children have been published, and those for esophageal strictures are generally used. For esophageal strictures, TTS balloon dilation is performed once every three weeks for three to five sessions till the desired endpoint (rule of 3 times the luminal diameter, 15 mm luminal diameter or symptomatic relief) is achieved.^3,4^ Some adult studies have shown that gastric outlet stenosis dilation to 12–15 mm luminal diameter results in significant improvement in symptoms, but these dilations generally should not occur beyond 3 mm per session due to higher risk of perforation. Therefore, repeat dilations are often needed, even as frequently as every 5–14 days, particularly when there is a severe narrowing and/or significant resistance during dilation.^5–7^ In addition, endoscopic intralesional corticosteroids and radial electroincisional therapy have been attempted in a few children with gastric strictures following corrosive ingestion to limit the need for subsequent interventions.^8^

In a series of 14 children with GOO, 6 responded with favorable outcomes to TTS balloon dilation alone, while 8 needed surgery.^9^ In a 20-year retrospective survey of 177 endoscopic balloon dilation in 72 adults with benign GOO, all 18 patients with postoperative strictures had symptomatic resolution and avoided surgery.^7^ Kochhar et al ^4^ reported a survey of 306 adult patients, of whom 264 underwent TTS balloon dilation. Caustic or medication-induced obstruction was more refractory to TTS balloon dilation compared with peptic ulcer-related obstruction.^4^ Depending on the causative factor, TTS balloon dilation can render surgery unnecessary in nearly 70% to 92.4% of adults with benign GOO. TTS balloon dilation offers several advantages over surgery for children with GOO and should be considered prior to surgery if the expertise is available. Complications of TTS balloon dilation in adults include perforation 2.6% and usually self-limited bleeding 1.3%.^4,7^ Complications are more likely with longer, multiple, deformed/eccentric/angular gastroduodenal strictures, and with those resulting from caustic ingestions.^4^ Complications of surgery include dumping syndrome, anemia, weight loss, pneumonia, wound infection, postoperative bleeding, anastomotic breakdown, anastomotic stricture, and even death in adult patients, who have more comorbidities.

GOO commonly manifests as persistent, nonbilious emesis, and may not display significant abdominal distention or visible gastric peristalsis on physical examination if much of the stomach has been resected in a prior surgery, as in the patient. Upper gastrointestinal contrast study before endoscopy is helpful to identify the degree of stenosis, site, shape (straight versus angular or deformed), and length of the GOO; it will also enable the providers to choose the optimal sized endoscopes and starting balloon size and exclude any anatomic complications from the GOO.

The authors have provided a comprehensive list of the etiologies of gastric stenoses in children.^1^ It is unclear if the stricture in this case was due to ischemia from PAN, an anastomotic stricture from the gastrectomy, or a combination of both. Details of the surgery done including laparoscopic versus open gastrectomy, type of gastrectomy, anastomotic techniques (staplers and manual suturing), and postoperative medical therapy including acid suppression or systemic steroid use may shed additional light on the development of the gastric stricture and subsequent resolution.

Congenital causes of spontaneous gastric perforation in differing age groups from premature newborns to adolescents include gastric volvulus, diaphragmatic hernia with strangulation, duodenal atresia, and duplication; acquired causes comprise of caustic (usually acidic) or foreign body (button battery, sharp objects, and magnets) ingestion, peptic ulcer, stress (burns, spinal cord injuries, and Curling’s ulcer), infection (rotavirus, norovirus, cytomegalovirus, tuberculosis, strongyloidosis, coronavirus 19, and fungal), trauma (blunt, physical abuse, and cardiopulmonary resuscitation), and iatrogenic (medications like non-steroidal anti-inflammatory drugs, nasogastric tube placement, surgery—gastrostomy tube migration, and postpyloromyotomy). ^10^ There is a broader range of rare causes that encompasses systemic vasculitides, connective tissue disorders, SLC2A10 mutation causing arterial tortuosity syndrome, eosinophilic gastroenteritis, anorexia nervosa, and neoplastic tumors. Spontaneous small and large bowel perforations from ischemic necrosis and ulceration have been described in a few children with PAN.^11^ However, this would be the only report in the pediatric literature of a gastric perforation in a child with PAN.

If one of the common causes of gastric perforation cannot be elicited from the history or physical examination, rare disorders causing gastric ischemia should be diligently sought. Pediatric surgeons may need to inspect the small and medium sized vasculature in the visceral organs, especially the liver, during surgical repair of the gastric perforation.^12^ It is important to scrutinize the gross pathology of the resected specimens as well as the histopathology of the gastric biopsies in deeper levels.

Angiographic studies may be indicated depending on the overall clinical picture, as in this patient.

PAN presenting with bowel perforation usually has a high mortality rate in children.^11^ It is highly commendable that this patient responded to timely surgical and medical interventions, including TTS balloon dilation.
